# Impact of participation in sports safety education on sports injuries, sports safety awareness, and sports activity habits among young Korean athletes

**DOI:** 10.1097/MD.0000000000041589

**Published:** 2025-02-21

**Authors:** Jeonga Kwon, Jusun Jang

**Affiliations:** a Department of Elementary Education, College of First, Korea National University of Education, Cheongju-si, Republic of Korea; b Department of Sports for All, College of Arts, Cheongju University, Cheongju-si, Republic of Korea.

**Keywords:** sports activity habits, sports injuries, sports safety awareness, sports safety education, youth athletes

## Abstract

Safety education is important for young athletes because it reduces injuries and serves as a stepping stone to becoming a professional athlete. Mandatory safety education and legislation for sports players are being discussed, but no progress has been made thus far. Therefore, this study investigated how young Korean athletes’ participation in sports safety education is related to sports injuries, sports safety awareness, and sports activity habits. We sourced all the data of 3262 professional athletes aged 13 to 18 years from the 2019 Sports Safety Accident Survey. We analyzed the data using SPSS for Windows (version 23.0; IBM Corp., Armonk, NY). Frequency analysis, chi-square analysis, and multivariate logistic regression were performed. The results revealed that the more athletes participate in safety education, the less likely they are to have sports injuries and the more likely they are to develop safety awareness and beneficial activity habits such as managing accidents. Additionally, those who participate in safety education are more likely to carry enough water to stay hydrated during exercise than those who do not. Overall, the results suggest that safety education should be emphasized for athletes from a young age, as it helps prevent injuries and improve performance. A governance-based safety education system must be established so young athletes can participate in safety education. Furthermore, safety education must be regular and sports-specific.

## 1. Introduction

Maslow^[1]^ defined safety as a basic need for survival. Followed by the need for food, clothing, and shelter, safety is a need 1 must meet to survive. Safety needs follow physiological needs, indicating that safety is important in life^[2]^; life is restricted if safety needs are not met. The Korean Ministry of Education^[3]^ has expanded school safety education into 7 domains (disaster safety, daily safety, first aid, drug and cyber addiction, traffic safety, violence and personal safety, and occupational safety) and mandated training. In addition, the 2015 revised physical education curriculum comprehensively covers accidents that can occur during physical activities and methods to prevent them. Hence, the importance of physical activity for the healthy development of growing children is being emphasized, and sports safety education to ensure this is becoming important.^[3,4]^

Sports safety education helps athletes to develop the knowledge and understanding of sports safety, skills and abilities required to practice safe movements, and attitude and willingness to perform sports safely.^[5]^ In other words, sports safety education creates a safe environment for sports activities and cultivates the ability to respond quickly to accidents. Cultivating sports safety awareness and developing accident response skills helps athletes reduce injuries.^[4]^ In fact, safety education on sports concussions has shown that adolescent athletes understand safety and injury prevention after participating than before participating in the program.^[6]^

This education is essential for athletes who play sports for a living because it reduces the possibility of accidents and inculcates a healthy exercise culture. Through safety education, athletes can obtain the knowledge, skills, and attitudes required to prevent accidents, more effectively demonstrate their skills, and fully enjoy sports. Because sports involve competition, dangerous situations can occur while engaging in sports activities. Young athletes are particularly prone to injuries, negatively affecting their athletic skills and physical growth.^[7]^ Injuries are also one of the reasons young athletes quit sports. If injuries are reduced, young athletes can fully display their skills and become professional athletes.^[8]^ To prevent injuries and enjoy sports, they must develop healthy sports habits. Adolescence is a period when 1 undergoes physical and mental development and builds lifestyle habits, values, and standards. Habits formed during this period continue into adulthood.^[9]^ Therefore, young individuals should learn the knowledge, values, and attitudes required for safely engaging in sports activities and form healthy sports habits to reduce injuries.^[5]^

Sports safety education is receiving widespread scholarly attention, given its importance. Lee^[5]^ conducted a meta-analysis on the effectiveness of safety education programs for school-age children and found a large-sized effect on students’ safety knowledge, safety practice behavior, and safety attitude. Choo^[10]^ implemented a marine leisure sports safety education program for college students using virtual reality and found that the participating group had greater safety awareness than the nonparticipating group. Lee et al^[11]^ provided cardio-pulmonary resuscitation training to badminton players and found that their knowledge increased with more training sessions, emphasizing the need for regular safety training. And Abernethy et al^[12]^ conducted a systematic review of strategies to prevent injuries in adolescents participating in sports activities. Their findings highlighted that pre-exercise safety education was a key factor in reducing the risk of injuries among youth during sports. Overall, the literature suggests that safety education helps form safety knowledge, skills, and attitudes, enabling individuals to engage in physical activities safely.

Regardless of how good athletes are, the average period of showing their skills is approximately 3 to 4 years in their 30s. During the time they are injured or a decline in their performance, they are dropped to a reserve player position, which prompts consideration of retirement.^[13]^ It may vary depending on the sport, but athletes consider retirement at 30 years of age, and in the case of Korean athletes, they retire in their 20s to 30s.^[14]^ There are various reasons why athletes retire, but 1 reason is unexpected injuries. This also applies to youth athletes, and sports safety education prevents injuries in student athletes, allowing them to grow into professional athletes. Furthermore, safety can be seen as playing an important role in career decisions.

The effectiveness of safety education can be achieved only when the direction of safety education is provided in accordance with the characteristics of the target audience. Sports safety education programs with different contents and levels should be developed depending on the life stage, such as children, adolescents, adults, and older adults, or according to aspects such as recreational sports participants and elite sports athletes.^[5–10]^ Adolescence is a time when people put what they have learned into practice and make it a habit, so learning is quick and effective. Accordingly, to prevent injuries and sports safety accidents among youth athletes, sports safety education programs tailored to their characteristics must be developed and disseminated. However, to establish an evidence base for the development of safety education programs that consider the characteristics of youth sports players, it is necessary to first examine in detail the extent to which participation in safety education affects sports injuries, sports safety awareness, and sports activity habits. In summary, although the effect of safety education is great, there is no suitable program for safety and accident education for youth sports players. To establish an evidence base for constructing a sports safety education program for youths, it is necessary to examine the impact of youth participation in sports safety education on young athletes’ knowledge, skills, and attitudes.

Safety education must be provided to young athletes who intend to engage in sports activities for a living because it reduces injuries and serves as a stepping stone to becoming professional athletes. Accordingly, mandatory safety training and legislation for sports players are being discussed, but no progress has been made.^[15]^ Therefore, we aimed to present objective data for developing accident prevention measures and implementing safety education legislation. This study investigated how young athletes’ participation in safety education is associated with sports injuries, sports safety awareness, and sports activity habits. Our results can provide insight into preventing accidents among young athletes, establishing a safety education system, and revitalizing safety education. Therefore, the hypotheses of this study are as follows. Participation in in sports safety education among Korean athletes will be significantly associated with the prevention of sports injuries. Participation in sports safety education among Korean athletes will be significantly associated with increased sports safety awareness. Participation in sports safety education will be significantly associated with the development of positive sports related habits.

## 2. Methods

### 2.1. Study population and data collection

The data used in this study were collected from the 2019 Sports Safety Accident Survey conducted by the Korea Sports Safety Foundation (https://www.sportsafety.or.kr). This survey is conducted to identify the types of accidents that occur during sports activities and establish prevention measures. The questionnaire is a validated questionnaire tool regarding sports injuries, sports safety awareness, and sports activity habits. The target demographic is recreational and professional athletes aged 13 years or older living in Korea. The Korea Sports Safety Foundation conducted 3 surveys between September 2019 and December 2019. After receiving training from the Korea Sports Safety Foundation, interviewers met one-on-one with the participants to explain the questionnaire and obtain consent. The survey participants responded by completing a paper questionnaire. Additionally, an assurance to protect personal information and ensure confidentiality was written at the top of the questionnaire. The survey was conducted among 11,745 individuals who had experienced injuries during sports activities in 2019, comprising 7725 recreational and 4020 professional athletes. The participants answered questions related to sports safety on a self-report form. The Sports Safety Foundation publishes information on its website for its data to be used for research. Identifying information, such as participants’ name, phone number, and email address, is deleted. Each participant is assigned a number, and data are uploaded to the website. We downloaded these data and did not use them for any purpose other than research. All data of 3262 professional athletes aged 13 to 18 were used. This is the number of professional athletes among the 13 to 18 aged participants. Because we used secondary data that did not include identifying information, such as name, telephone number, home address, and social security number, ethical approval was not required. Nonetheless, this study was conducted in accordance with the principles outlined in the Declaration of Helsinki.

### 2.2. Variables and measurements

The study variables were participation in safety education, sports injuries, sports safety awareness, and sports activity habits. Safety education participation was measured by asking respondents, “Outside of the regular curriculum (elementary, middle, and high school education), have you ever received safety education to prepare you for accidents and injuries during sports activities?” Respondents could answer this question with 1 (yes) or 2 (no). We used their responses without making any modifications. In Korea, out-of-school sports safety education for young Korean athletes is provided by associations and safety education institutions, and safety education is also offered in schools. However, it is not systematic because non-athletes participate in it and safety education appropriate for the sport is not covered. Sports injuries were measured on the basis of 2 aspects: the severity of sports injuries and the number of injuries sustained. To determine the severity of sports injuries, the respondents were asked, “How severe was your sports injury?” Respondents could answer with 1 (“severe injury”), 2 (“moderate injury”), or 3 (“slight injury”). We used these responses without making any modifications. The number of sports injuries sustained was measured by asking, “In the past year, how many injuries did you sustain while playing sports?” Respondents were asked to write their answers subjectively. We categorized their responses as 1, 2, 3, 4, 5, or more times.

Sports safety awareness was measured on the basis of 3 aspects: awareness of how to prevent sports accidents, awareness of how to handle sports accidents, and awareness of sports safety rules. Awareness of how to prevent sports accidents was measured by asking respondents, “How much do you know about preventing sports accidents?” Awareness of how to handle sports accidents was measured by asking, “How much do you know about handling sports accidents?” Awareness of sports safety rules was assessed by asking, “How much do you know about the safety rules for the sport you play?” For all 3 questions, respondents rated their answer on a 5-point Likert scale: 1, I do not know at all; 2, I do not know a lot; 3, I usually know; 4, I know a lot; and 5, I know very well. We categorized these responses as “I am not aware” or “I am aware.”

The sports activity habits were performing warm-up exercises, verifying the defects and safety of surrounding facilities, participating in exercises according to one’s fitness level, carrying enough water to stay hydrated, drinking water during rest times, wearing the proper clothes and safety equipment, assessing environmental factors, and performing finishing exercises. Whether 1 performed warm-up exercises was measured by asking, “Do you warm up before a workout?” Whether 1 checked the defects and safety of surrounding facilities was measured by asking, “Do you verify the defects and safety of surrounding facilities before you start exercising?” Whether 1 participated in exercises according to one’s fitness level was assessed by asking, “Do you know your fitness level before you start exercising, and do you exercise accordingly?” Whether 1 carried enough water was measured by asking, “Do you carry enough water to stay hydrated?” Whether 1 drank water during rest times was measured by asking, “Do you drink water during designated rest times?” Whether 1 wore the proper clothes and safety equipment was assessed by asking, “Do you wear the proper clothing and safety equipment for your sport?” Whether 1 evaluated environmental factor was assessed by asking, “Do you consider environmental factors, such as weather and signs of disasters, before exercising?” Whether 1 performed finishing exercises was measured by asking, “Do you do finishing exercises?” For all questions, respondents rated their answer on a 5-point Likert scale: 1, “not at all”; 2, “rarely”; 3, “sometimes”; 4, “often”; and 5, “always.” We categorized these responses as “no” or “yes.”

The covariates were sex, the frequency of sports participation, and the extent of sports technology implementation. Sex was classified as male or female. The frequency of sports participation was measured by asking, “In the past year, how often have you participated in a sport?” The response options were 1 (daily), 2 (4–5 times a week), 3 (2–3 times a week), 4 (once a week), 5 (2–3 times a month), 6 (once a month), 7 (once every 2–3 months), 8 (once every 4–5 months), 9 (once every 6 months), and 10 (irregularly). We categorized these responses as “daily,” “4 to 5 times a week,” “1 to 3 times a week,” “1 to 3 times a month,” “once every 2 to 6 months,” or “never.” The extent of sports technology implementation was measured by asking, “To what extent can you implement technology in your sport?” The response options were 1 (no extent), 2 (small extent), 3 (moderate extent), 4 (good extent), and 5 (complete extent). We used these responses without making any modifications. The questionnaire used in this study is summarized in Table 1.

**Table 1:** The questionnaire used in this study summarized each item.

Variables	Items	Contents
Safety education participation	Safety education participation	Outside of the regular curriculum (elementary, middle, and high school education), have you received safety education to prepare you for accidents and injuries during sports activities?
Sports injuries	Severity of sports injuries	How severe was your sports injury?
	The number of sports injuries	In the past year, how many injuries did you sustain while playing sports?
Sports safety awareness	Awareness of how to prevent sports accidents	How much do you know about preventing sports accidents?
	Awareness of how to handle sports accidents	How much do you know about handling sports accidents?
	Awareness of sports safety rules	How much do you know about the safety rules for the sport you play?
Sports activity habits	Warm-up exercises	Do you warm up before a workout?
	Verifying the defects	Do you verify the defects and safety of surrounding facilities before you start exercising?
	Safety of surrounding facilities	Do you verify the safety of surrounding facilities before you start exercising?
	Participating in exercises according to one’s fitness level	Do you know your fitness level before you start exercising, and do you exercise accordingly?
	Carrying enough water to stay hydrated	Do you carry enough water to stay hydrated?
	Drank water during rest times	Do you drink water during the designated rest times?
	Wearing the proper clothes and safety equipment	Do you wear proper clothing and use safety equipment for your sport?
	Assessing environmental factors	Do you consider environmental factors, such as weather and signs of disasters, before exercising?
	Performing cool down	Do you finish your work out session with a cooling down routine?

### 2.3. Data analysis

All statistical analyses were performed using SPSS for Windows (version 23.0; IBM Corp., Armonk, NY). First, a frequency analysis was performed to determine the characteristics of the study population. Second, we conducted chi-square analyses to determine differences in each variable according to respondents’ participation in safety education. Third, we performed multivariate logistic regression analysis to determine the association of young athletes’ participation in safety education with sports injuries, sports safety awareness, and sports activity habits. We verified and confirmed the collinearity among variables. Statistical significance was set at *P* < .05.

## 3. Results

### 3.1. Study population characteristics

Table 2 presents the characteristics of the 3262 respondents. The majority of the participants participated in sports activities on a daily basis.

**Table 2:** Study population characteristics (n = 3262).

Variable		Mean ± standard deviation or frequency (%)
Sex	Male	2476 (75.9%)
	Female	786 (24.1%)
Frequency of sports participation	Daily	1651 (50.6%)
	4–5 times a week	1496 (45.9%)
	1–3 times a week	82 (2.5%)
	1–3 times a month	4 (0.1%)
	Once every 2–6 months	17 (0.5%)
	Never	12 (0.4%)
Extent of sports technology implementation	No	125 (3.8%)
	Small	286 (8.8%)
	Moderate	1003 (30.7%)
	Good	860 (26.4%)
	Complete	988 (30.3%)
Safety education participation	Participated	2090 (64.1%)
	Did not participate	1172 (35.9%)
Severity of sports injuries	Severe	430 (13.2%)
	Moderate	1137 (34.8%)
	Slight	1695 (52.0%)
Number of sports injuries sustained	1	934 (28.6%)
	2	753 (23.1%)
	3	538 (16.5%)
	4	258 (7.9%)
	≥5	779 (23.9%)
Performing warm-up exercises	No	163 (5.0%)
	Yes	3099 (98.0%)
Verifying the defects and safety of surrounding facilities	No	892 (27.3%)
	Yes	2370 (72.7%)
Participating in exercises according to one’s fitness level	No	517 (15.8%)
	Yes	2745 (84.2%)
Carrying enough water to stay hydrated	No	505 (15.5%)
	Yes	2757 (84.5%)
Drinking water during rest times	No	599 (18.4%)
	Yes	2663 (81.6%)
Wearing the proper clothes and safety equipment	No	615 (18.9%)
	Yes	2647 (81.1%)
Assessing environmental factors	No	1327 (40.7%)
	Yes	1935 (59.3%)
Performing finishing exercises	No	376 (11.5%)
	Yes	2886 (98.5%)
Awareness of how to prevent sports accidents	I am not aware	994 (30.5%)
	I am aware	2268 (69.5%)
Awareness of how to handle sports accidents	I am not aware	1118 (34.3%)
	I am aware	2144 (65.7%)
Awareness of sports safety rules	I am not aware	892 (27.3%)
	I am aware	2370 (72.7%)

### 3.2. Differences in variables based on safety education participation

Table 3 presents the chi-square analyses results. The variables including sex (χ^2^ = 19.237, *P* < .001), severity of sports injuries (χ^2^ = 6.943, *P* = .031), number of sports injuries sustained (χ^2^ = 16.209, *P* = .003), performing warm-up exercises (χ^2^ = 6.684, *P* = .012), verifying the defects and safety of surrounding facilities (χ^2^ = 16.433, *P* < .001), participating in exercises according to one’s fitness level (χ^2^ = 33.877, *P* < .001), carrying enough water to stay hydrated (χ^2^ = 41.116, *P* < .001), drinking water during rest times (χ^2^ = 22.912, *P* < .001), wearing the proper clothes and safety equipment (χ^2^ = 19.259, *P* < .001), assessing environmental factors (χ^2^ = 20.015, *P* < .001), awareness of how to prevent sports accidents (χ^2^ = 71.785, *P* < .001), awareness of how to handle sports accidents (χ^2^ = 95.823, *P* < .001), and awareness of sports safety rules (χ^2^ = 90.996, *P* < .001) differed significantly on the basis of safety education participation.

**Table 3:** Differences in variables based on safety education participation.

Variable		Participation in safety education (n = 2090)	Nonparticipation in safety education (n = 1172)	χ^2^ (*P*)
Sex	Male	1535 (73.4%)	941 (80.3%)	19.237 (<.001***)
	Female	555 (26.6%)	231 (19.7%)	
Severity of sports injuries	Severe	284 (13.6%)	146 (12.5%)	6.943 (.031*)
	Moderate	756 (36.2%)	381 (32.5%)	
	Slight	1050 (50.2%)	645 (55.0%)	
Number of sports injuries sustained	1	598 (28.6%)	336 (28.7%)	16.029 (.003**)
	2	445 (21.3%)	308 (26.3%)	
	3	342 (16.4%)	196 (16.7%)	
	4	182 (8.7%)	76 (6.5%)	
	≥5	523 (25.0%)	256 (21.8%)	
Performing warm-up exercises	No	89 (4.3%)	74 (6.3%)	6.684 (.012*)
	Yes	2001 (95.7%)	1098 (93.7%)	
Verifying the defects and safety of surrounding facilities	No	522 (25.0%)	370 (31.6%)	16.433 (<.001***)
	Yes	1568 (75.0%)	802 (68.4%)	
Participating in exercises according to one’s fitness level	No	273 (13.1%)	244 (20.8%)	33.877 (<.001***)
	Yes	1817 (86.9%)	928 (79.2%)	
Carrying enough water to stay hydrated	No	260 (12.4%)	245 (20.9%)	41.116 (<.001***)
	Yes	1830 (87.6%)	927 (79.1%)	
Drinking water during rest times	No	333 (15.9%)	266 (22.7%)	22.912 (<.001***)
	Yes	1757 (84.1%)	906 (77.3%)	
Wearing the proper clothes and safety equipment	No	347 (16.6%)	268 (22.9%)	19.259 (<.001***)
	Yes	1743 (83.4%)	904 (77.1%)	
Assessing environmental factors	No	790 (37.8%)	537 (45.8%)	20.015 (<.001***)
	Yes	1300 (62.2%)	635 (54.2%)	
Performing finishing exercises	No	224 (10.7%)	152 (13.0%)	3.733 (.059)
	Yes	1866 (89.3%)	1020 (87.0%)	
Awareness of how to prevent sports accidents	I am not aware	530 (25.4%)	464 (39.6%)	71.785 (<.001***)
	I am aware	1560 (74.6%)	708 (60.4%)	
Awareness of how to handle sports accidents	I am not aware	589 (28.2%)	529 (45.1%)	95.823 (<.001***)
	I am aware	1501 (71.8%)	643 (54.9%)	
Awareness of sports safety rules	I am not aware	455 (21.8%)	437 (37.3%)	90.996 (<.001***)
	I am aware	1635 (78.2%)	735 (62.7%)	

* *P* < .05, assessed through chi-square analyses.

** *P* < .01, assessed through chi-square analyses.

*** *P* < .001, assessed through chi-square analyses.

### 3.3. Relationship between safety education participation and sports injuries

Table 4 shows the multivariate logistic regression results analyzing the relationship between safety education participation and sports injuries. The results for the number of sports injuries sustained were statistically significant. When young athletes participated in safety education, the odds ratio (95% confidence interval [CI], *P*-value) of getting injured 2 times in a year was 0.717 (0.577–0.891, *P* = .003). In other words, if young athletes participated in safety education, the number of sports injuries they sustained would have likely decreased. Conversely, the results for the severity of sports injuries were not statistically significant.

**Table 4:** Relationship between safety education participation and sports injuries.

Variable		Odds ratio	95% confidence interval	*P*-value
Severity of sports injuries	Slight (n = 430)	Reference		
	Moderate (n = 1137)	1.162	0.984–1.372	.076
	Severe (n = 1695)	1.149	0.909–1.453	.246
Number of sports injuries sustained	1 (n = 934)	0.865	0.699–1.070	.181
	2 (n = 753)	0.717	0.577–0.891	.003**
	3 (n = 538)	0.826	0.650–1.048	.115
	4 (n = 258)	1.187	0.866–1.626	.287
	≥ 5 (n = 779)	Reference		

** *P* < .01; assessed through multivariate logistic regression analysis.

### 3.4. Relationship between safety education participation and sports safety awareness

Table 5 shows the multivariate logistic regression results analyzing the relationship between safety education participation and sports safety awareness. The results were statistically significant for awareness of how to handle sports accidents and sports safety rules awareness. When 1 participated in safety education, the odds ratios (95% CIs, *P*-value) of being aware of how to handle sports accidents and being aware of sports safety rules were 1.609 (1.277–2.027, *P* < .001) and 1.644 (1.369–1.973, *P* < .001), respectively. In other words, young athletes who received safety education were more likely to be aware of sports safety rules and how to handle sports accidents than those who did not. The results for the awareness of how to prevent sports accidents were not statistically significant.

**Table 5:** Relationship between safety education participation and sports safety awareness.

Variable		Odds ratio	95% confidence interval	*P*-value
Awareness of how to prevent sports accidents	I am not aware (n = 994)	Reference		
	I am aware (n = 2268)	0.969	0.757–1.241	.804
Awareness of how to handle sports accidents	I am not aware (n = 1118)	Reference		
	I am aware (n = 2114)	1.609	1.277–2.027	<.001***
Sports safety rules awareness	I am not aware (n = 892)	Reference		
	I am aware (n = 2370)	1.644	1.369–1.973	<.001***

*** *P* < .001, assessed through multivariate logistic regression analysis.

### 3.5. Relationship between safety education participation and sports activity habits

Table 6 shows the multivariate logistic regression results analyzing the relationship between safety education participation and sports activity habits. The results were statistically significant only for carrying enough water to stay hydrated. When 1 participated in safety education, the odds ratio (95% CI, *P*-value) of carrying enough water to stay hydrated was 1.409 (1.100–1.804, *P* = .007). In other words, young athletes who received safety education were more likely to carry enough water to stay hydrated during sports activities than those who did not.

**Table 6:** Relationship between safety education participation and sports activity habits.

Variable		Odds ratio	95% confidence interval	*P*-value
Performing warm-up exercises	No (n = 163)	Reference		
	Yes (n = 3099)	1.041	0.732–1.482	.822
Verifying the defects and safety of surrounding facilities	No (n = 892)	Reference		
	Yes (n = 2370)	1.062	0.890–1.267	.504
Participating in exercises according to one’s fitness level	No (n = 517)	Reference		
	Yes (n = 2745)	1.179	0.932–1.492	.170
Carrying enough water to stay hydrated	No (n = 505)	Reference		
	Yes (n = 2757)	1.409	1.100–1.804	.007**
Drinking water during rest times	No (n = 599)	Reference		
	Yes (n = 2663)	1.057	0.839–1.331	.638
Wearing the proper clothing and safety equipment	No (n = 615)	Reference		
	Yes (n = 2647)	1.107	0.910–1.347	.311
Assessing environmental factors	No (n = 1327)	Reference		
	Yes (n = 1935)	1.121	0.957–1.312	.157
Performing finishing exercises	No (n = 376)	Reference		
	Yes (n = 2886)	0.929	0.728–1.185	.552

** *P* < .01, assessed through multivariate logistic regression analysis.

## 4. Discussion

Our results showed that the more young Korean athletes participate in safety education, the less likely they are to sustain a sports injury and the more likely they are to form sports activity habits and be aware of sports safety. First, we found that safety education tailored to the sport of choice is not adequately provided. Safety education is offered to most school students, whether they are athletes. Although this education is comprehensive, it is insufficient to prevent injuries during sports activities.^[16]^ In addition to safety education for athletes to prevent injuries, safety education related to life, driving, violence, and drugs is provided. Additionally, since the situation and location of injury are different for each type of sport, it is necessary to provide sports safety education tailored to the specific sport. Consequently, young athletes participate in safety education specialized for their sports at facilities outside school, such as public institutions, associations, and clubs.^[17]^ However, athletes, coaches, managers, and player managers cannot properly participate in safety education tailored to the type of sport for because of the lack of facilities, accessibility, information about such facilities, or poorly designed programs.^[17]^

This has led to low participation in safety education outside school. Therefore, a governance-based safety education system must be established in Korea to create an environment where young athletes can easily receive safety education. This will encourage young Korean athletes to participate in sports safely by allowing them to participate in safety education appropriate to their sport.

Second, we found that youth athletes’ participation in safety education is significantly related to reduced sports injuries. This result is consistent with findings of several studies that found that young athletes’ participation in safety education effectively prevents injuries.^[12,18]^ This suggests that sports safety education plays a mediating role in helping to prevent injuries among youth athletes.^[19]^ Eliminating injuries from sports activities is impossible; however, they can be predicted and prevented. The number and severity of injuries can be reduced by providing safety education tailored to the sport 1 plays.^[20]^ In fact, Rössler et al^[21]^ conducted a meta-analysis on the effectiveness of adolescent participation in sports injury prevention programs. Their results showed that prevention programs that focused on specific injuries were effective in reducing injuries among adolescents. The Sports and Exercise Safety (LiVE) program (2006 to the present) aims to reduce sport injuries in Finland.^[22]^ Thus, the website provides various information (safety education and injury prevention strategies) for young athletes, coaches, instructors, team managers, and families to foster a culture of sports coaching and training that promotes health and safety.^[22]^ Once an injury occurs, the injured area becomes vulnerable and may be susceptible to other injuries.^[23]^ Of the study participants, 12 (0.4%) student athletes had not participated in any sporting activities in the past year. Many reasons exist for athletes not participating in sports activities, including lack of ability, social pressure, and injury.^[24]^ Among these reasons, injuries can lead athletes to quit the sport entirely, even for players who have performed well, so caution is needed in these cases.^[25]^ Our results suggest that it is necessary to provide tailored safety education to athletes from a young age so they can safely participate in sports activities and minimize injuries. Systematic safety education centered on one’s sport that allows young athletes to predict and prevent injuries is needed.

Third, we found that participation in safety education is significantly related to forming sports safety awareness. More specifically, we found that participating in safety education strongly correlates with being aware of safety rules and knowing how to handle sports accidents. This result aligns with findings of several studies showing that people who participate in sports safety education have greater safety awareness than those who do not.^[11,26,27]^ Park et al^[26]^ found that scuba divers receiving safety training have better safety knowledge and ability to respond to emergencies than those who do not. Moreover, they exhibit better compliance with safety rules than those who do not receive safety training. Nurjayanti et al^[27]^ taught rest, ice, compression, and elevation therapy for ankle sprains through role-play. After this training, students improved their awareness of related first-aid methods and coping skills. Lee et al^[11]^ revealed that the more frequently 1 participates in cardio-pulmonary resuscitation training, the higher one’s related safety knowledge and awareness. They also found a strong correlation between the frequency of participation in safety training and safety awareness. This supports the idea that participation in safety education increases young athletes’ sports safety awareness. By teaching how to make quick and accurate decisions in dangerous situations, safety awareness reduces future risks and empowers an individual to avoid crises.^[28]^ Additionally, safety awareness gained through participation in safety education can positively affect safety behavior. The more safety knowledge 1 has, the more likely 1 will exhibit safety behaviors.^[29]^ However, knowledge obtained through safety education often requires revision because the effect of training is not permanent; as time passes, the learned content is often forgotten.^[30]^ To maintain and increase safety knowledge, safety education should be provided regularly to young athletes rather than once.

Fourth, we found that participation in safety education positively affects the formation of sports activity habits. More specifically, we found that athletes participating in safety education are more likely to carry enough water to stay hydrated during exercise. This result aligns with findings of studies that found that safety education significantly affects attitudes, behaviors, and habits that allow 1 to participate in sports activities safely.^[5,31]^ Sports safety habits are important prerequisites for continued participation in sports. Exhibiting sports safety behaviors regularly, such as doing warm-up and finishing exercises, carrying enough water, checking one’s body temperature, taking adequate rest, verifying the facility’s safety, and wearing the proper clothing and protective equipment, can prevent sports accidents. These habits can be an effective method to reduce the likelihood of injuries.^[20,32–34]^ Moreover, these habits are strongly related to athletic performance. Practicing safety attitudes and behaviors improves exercise commitment and the intention to continue exercising. Conversely, failure to implement safety behaviors has negatively affected athletic performance.^[35,36]^ The more safety education sessions an individual attends, the more likely they are to practice safety behaviors and implement safety education content, lowering the incidence of accidents.^[5]^ Young athletes can reduce the likelihood of injury by consistently applying safety knowledge learned from safety education to their games. Given that safety education plays a major mediating role that helps improve athletes’ performance, it must be emphasized to foster sports activity habits in young athletes.

### 4.1. Practical implications of the findings

This study confirmed that young athletes’ participation in sports safety education will likely reduce the possibility of sports injuries, form sports activity habits, and increase their awareness of sports safety. However, only 64.1% of young Korean athletes participate in sports safety education, necessitating increased participation. First, as stated earlier, a governance-based safety education system should be established. Owing to the lack of facilities that provide proper safety education, related information, and effective programs, it is necessary to support young athletes’ participation in safety education. Establishing a governance-based safety education system can help athletes participate regularly in safety education programs.^[2,37]^ This can be realized through the cooperation of the central government, local governments, and private organizations. For example, in Germany, swimming education is compulsory in all regions with the goal of “survival” in the water, and local governments and schools work together to ensure straightforward swimming education.^[38]^ Additionally, Germany strives for safety education by fostering safety professionals who are sports experts. Specifically, Bayern Leberkusen employs BaySecur, which contributes to the training of sports safety professionals by providing safety training and first-aid education.^[39,40]^ Additionally, a permanent safety education center can be established solely for athletes by collaborating with the local government and the governmental body in charge of sports.^[40]^ Equipment support can be received by signing a memorandum of understanding with a local company to provide hands-on safety education. The state must enter into a business agreement with an organization that will systematically take charge of safety education for adolescent athletes.^[41]^ In addition, the state needs to establish a policy to decentralize safety education centers to local governments so that adolescent athletes can participate in safety education.^[42]^

Second, safety education provided to athletes must be tailored to the sport they play. Various factors cause young athletes to get injured during sports activities, such as their sex, low physical ability, incorrect coaching, previous injuries, and equipment problems. These factors and the area of injury also differ on the basis of the sport.^[43,44]^ Because sports accidents differ on the basis of the sport, they can be prevented by providing safety education tailored to the sport and eliminating the factors that cause them.^[45]^ For example, policy-disallowing body checking in youth ice hockey has led to a > 50% reduction in injuries, and rules limiting contact practice in youth American football have significant potential for injury prevention.^[20]^ There is evidence to support the use of bracing and taping in elite sports to reduce the risk of recurrent ankle sprain injury but not to prevent primary injury, and wrist guards are protective against sprain injuries during snowboarding.^[20]^ At this time, repetitive and periodic safety education centered on practical skills can be a good educational method to increase educational effectiveness.^[2,5]^ Studies by Kwon^[2]^ and Lee^[5]^ also revealed that teaching through role-playing and learning in simulated situations is more effective than theory. Accordingly, if the association hires retired athletes from each sport as instructors and operates a case-based, experience-oriented safety education program, it will be possible to operate customized safety training optimized for the sport.

### 4.2. Limitations and suggestions for future research

This study has some limitations. First, a cross-sectional study with a secondary analysis of the 2019 Sports Safety Accident Survey data was performed. We used data on young athletes’ participation in safety education, sports injuries, sports activity habits, and sports safety awareness. However, we could not determine causal relationships between these variables. Second, the data collected were self-reported by the respondents, which might have resulted in measurement errors. Third, the survey was conducted in 2019; thus, we used predictive models with data collected 4 years ago. Although the data are data, the Sports Safety Accident Survey in Republic of Korea is conducted every 5 years, so the next updated survey will be conducted between September 2024 and December 2024. Then, these data will be available to researchers in late 2025. Moreover, although this survey was conducted in 2019, it has the advantage of providing basic data that can identify the status and causes of sports safety accidents and be used to suggest related policy directions. Therefore, despite the passage of time, research using the raw data from the 2019 Sports Safety Accident Survey is actively underway.^[46–48]^ Fourth, although the study population is large, it is not representative. Men are overrepresented, so the results may not be generalizable to all young athletes. Fifth, participation and nonparticipation in safety education were used to determine whether young athletes engaged in safety education inside and outside school. Owing to geographical conditions, the nonparticipation in safety education does not include the young players who received safety education at school from coaches or invited experts. Additionally, the results may have been influenced by the results of adolescents who did not receive safety education on accidents and injuries during sports activities outside of school, but did receive safety education related to this in their regular curriculum. Sixth, the cross-sectional studies can reveal a relationship but cannot assess causality. Therefore, well-trained epidemiology researchers believe that it is tangential and unnecessary to extensively discuss varied opinions or theories. Although a discussion of theories is lacking herein, we believe it is important in epidemiology reports to simply present the results because cause-and-effect relationships are not typically revealed. The failure to consider these details might have influenced the results of the chi-square analyses verification study. Nevertheless, this study is significant because it identified the relationship between young Korean athletes’ participation in sports safety education and sports injuries, sports safety awareness, and sports activity habits. Moreover, this study’s results can be used as a foundation for establishing policies that prevent sports accidents among young Korean athletes. They will also contribute to establishing a safety education system. This study confirmed that safety education is highly relevant in reducing sports injuries among young athletes. Besides safety education, there are likely more factors affecting sports injuries among young athletes. Accordingly, future research should explore factors influencing the reduction of sports injuries in young athletes.

## 5. Conclusion

Young Korean athletes’ participation in safety education is low. The more they participate in safety education, the less likely they are to sustain sports injuries and the more likely they are to form sports activity habits and be aware of sports safety. As safety education plays a major role in preventing injuries and improving athletic performance, it must be emphasized for athletes from a young age. Young Korean athletes cannot participate in safety education because of the lack of educational institutions or information about such programs. Therefore, establishing a governance-based safety education system and creating an environment where they can easily receive safety education tailored to their sport is crucial.

## Acknowledgments

The authors thank the study participants who generously volunteered to participate in the present study.

## Author contributions

**Writing – original draft:** Jeonga Kwon, Jusun Jang.

**Writing – review & editing:** Jeonga Kwon, Jusun Jang.

## References

[1] Maslow. Motivation and Personality. Harper; 1954.

[2] Kwon JA Park JJ. Elementary safety education awareness through big data. J Learner-Centered Curriculum Instr. 2020;20:1351–71.

[3] Korea Ministry of Education. Seven Safety Education Standards. Korea Ministry of Education; 2016.

[4] Korea Sports Safety Foundation. Comprehensive Report on the Status of Sports Safety Accidents; Korea Sports Safety Foundation; 2016.

[5] Lee SJ. Meta-analysis on the effects of safety education program school age children. J Learner-Centered Curriculum Instr. 2022;22:197–208.

[6] Elliott RJS Batiste O Hitto I Walker B Leary LD. Pediatric sport-related concussion education: effectiveness and long-term retention of the head safety in youth sports (HSYS) program for youth athletes aged 11–16. Cogent Educ. 2016;3:1256141.

[7] Lee IJ Lee SY Ha SH. Differences in sports safety awareness between recreational and elite soccer players. Korean J Phys Educ. 2021;60:45–54.

[8] Prieto-González P Martínez-Castillo JL Fernández-Galván LM Casado A Soporki S Sánchez-Infante J. Epidemiology of sports-related injuries and associated risk factors in adolescent athletes: an injury surveillance. Int J Environ Res Public Health. 2021;18:4857. 34063226 10.3390/ijerph18094857 PMC8125505

[9] Batista MB Romanzini CLP Barbosa CCL Blasquez Shigaki G Romanzini M Ronque ERV. Participation in sports in childhood and adolescence and physical activity in adulthood: a systematic review. J Sports Sci. 2019;37:2253–62. 31179841 10.1080/02640414.2019.1627696

[10] Choo JH. Verification of effectiveness of virtual reality (VR) program for safety education of marine leisure sport. J Mar Tour Res. 2022;15:265–80.

[11] Lee DJ Kim MK Oh DJ. The study about effect of CPR educational of badminton club member on knowledge, attitude, and safety consciousness. Korean J Sports Sci. 2018;27:657–67.

[12] Abernethy L Bleakley C. Strategies to prevent injury in adolescent sport: a systematic review. Br J Sports Med. 2007;41:627–38. 17496070 10.1136/bjsm.2007.035691 PMC2465167

[13] Eitzen DS Sage GH. Sociology of North American Sport, Madison, WI: Brown & Benchmark. Empirical Considerations. Soc Sport J. 1997;2:101–10.

[14] Lee YS. A study on retirement preparation and career support for national athlete in Korea. Korean J Sport Sci. 2008;19:136–45.

[15] An KJ Jang SW. A study on the necessity of mandatory safety education for ensuring safety in sports activities: focusing on the current law. Korean J Phys Educ. 2023;62:261–71.

[16] Cho IS Jung PW. A critical analysis of safety education in school. J Hum Rights Law-relat Educ. 2015;8:43–64.

[17] Korea Sports Safety Foundation. Comprehensive Report on the Status of Sports Safety Accidents. Korea Sports Safety Foundation; 2020.

[18] Black AM Meeuwisse DW Eliason PH Hagel BE Emery CA. Sport participation and injury rates in high school students: a Canadian survey of 2029 adolescents. J Safety Res. 2021;78:314–21. 34399928 10.1016/j.jsr.2021.06.008

[19] Goossens L De Ridder R Cardon G Witvrouw E Verrelst R De Clercq D. Injury prevention in physical education teacher education students: lessons from sports. A systematic review. Eur Phys Educ Rev. 2019;25:156–73.

[20] Emery CA Pasanen K. Current trends in sport injury prevention. Best Pract Res Clin Rheumatol. 2019;33:3–15. 31431273 10.1016/j.berh.2019.02.009

[21] Rössler R Donath L Verhagen E Junge A Schweizer T Faude O. Exercise-based injury prevention in child and adolescent sport: a systematic review and meta-analysis. Sports Med. 2014;44:1733–48. 25129698 10.1007/s40279-014-0234-2

[22] Ojala A Jussila AM Pasanen K Parkkari J. 743 Healthy athlete nationwide sport safety implementation case to sport clubs. Inj Prev. 2016;22:266–7.

[23] Caine D Maffulli N Caine C. Epidemiology of injury in child and adolescent sports: injury rates, risk factors, and prevention. Clin Sports Med. 2008;27:19–50, vii. 18206567 10.1016/j.csm.2007.10.008

[24] Crane J Temple V. A systematic review of dropout from organized sport among children and youth. Eur Phys Educ Rev. 2015;21:114–31.

[25] Jacobsson J Mirkovic D Hansson PO Lundqvist C Mann RH Tranaeus U. Youth athletes at Swedish sports high schools with an athletics specialism emphasize environmental support for injury risk management: a focus group study. BMJ Open Sport Exerc Med. 2023;9:e001527. 10.1136/bmjsem-2022-001527 PMC10186414 37200774

[26] Park HC Cho KJ. Correlation among knowledge of safety, compliance with safety rules, and ability to cope with emergency situations of scuba divers. Korean J Emerg Med Serv. 2015;19:35–49.

[27] Nurjayanti R Romadoni S. Educational influence with role play about first aid rice in sports injury ankle sprain on student knowledge school. J Inspirasi Kesehatan. 2023;1:58–64.

[28] Kum EH. The effects of simulation method using book art on safety knowledge and safety behaviors. Korea National University of Education, Chungcheongbuk-do, Repubilc of Korea. Master’s thesis. 2014.

[29] Lee YJ. Elementary school children’s experience of safety education and their knowledge, attitude, and behavior in safety. J Educ. 2018;38:327–47.

[30] Woollard M Whitfield R Smith A. Skill acquisition and retention in automated external defibrillator (AED) use and CPR by lay responders: a prospective study. Resuscitation. 2004;60:17–28. 15002485 10.1016/j.resuscitation.2003.09.006

[31] Kroshus E Baugh CM. Concussion education in US collegiate sport: what is happening and what do athletes want? Health Educ Behav. 2016;43:182–90. 26293460 10.1177/1090198115599380 PMC4761331

[32] Sabato TM Walch TJ Caine DJ. The elite young athlete: strategies to ensure physical and emotional health. Open Access J Sports Med. 2016;7:99–113. 27621677 10.2147/OAJSM.S96821 PMC5012846

[33] Foss KDB Le Cara E McCambridge T Hinton RY Kushner A Myer GD. Epidemiology of injuries in women’s lacrosse: implications for sport-, level-, and sex-specific injury prevention strategies. Clin J Sport Med. 2018;28:406–13. 28742608 10.1097/JSM.0000000000000458

[34] Jeffreys I. The warm-up. In Advanced Personal Training. Routledge; 2021. pp 129–42.

[35] Kraak W Coetzee L Kruger A Stewart R van Vuuren H. Knowledge and attitudes towards concussion in Western Province rugby union senior club rugby players. Int J Sports Med. 2019;40:825–30. 31574545 10.1055/a-0959-2113

[36] Moon GP Park SH Kim B. Influence of risk perceived and hedonic value of a swimming club members on safety attitude, exercise commitment & intention to continue exercise. Korean J Sport. 2020;18:261–70.

[37] Backe S Janson S Timpka T. Governance and implementation of sports safety practices by municipal offices in Swedish communities. Int J Inj Contr Saf Promot. 2012;19:163–9. 22126404 10.1080/17457300.2011.635212

[38] Youk HC. A Comparative study on swimming education in the elementary and middle school between Korea and Germany. Sports Sci. 2018;35:17–25.

[39] Kim IG Kwon HW Choi JH. Safety management network of sports facilities abroad and system status. J Digital Convergence. 2016;14:547–62.

[40] Ju SS Park SB. Establishment of local-based collaborative governance for development elementary school sport. J Korea Elem Educ. 2022;33:51–72.

[41] Gurgis JJ Kerr GA. Sport administrators’ perspectives on advancing safe sport. Front Sports Act Living. 2021;3:630071. 34169275 10.3389/fspor.2021.630071 PMC8217753

[42] Gao Y. The effect of online and offline sports safety education combined with MOOC platforms in physical education teaching in colleges and universities. Scalable Comput Pract Exp. 2023;24:585–96.

[43] Vanderlei FM Vanderlei L Bastos FN Netto Júnior J Pastre CM. Characteristics and associated factors with sports injuries among children and adolescents. Braz J Phys Ther. 2014;18:530–7. 25590445 10.1590/bjpt-rbf.2014.0059 PMC4311597

[44] Owoeye OB Palacios-Derflingher LM Emery CA. Prevention of ankle sprain injuries in youth soccer and basketball: effectiveness of a neuromuscular training program and examining risk factors. Clin J Sport Med. 2018;28:325–31. 29864071 10.1097/JSM.0000000000000462

[45] Nam SK Kim DH. The study on real condition of the safety accident and countermeasures for safety management of man’s artistic gymnasts. Korean J Sport. 2016;14:37–46.

[46] Kim RH Seo IH. A study on the importance of sports safety education, continuity of participation, and awareness of safety culture: focusing on sports leaders. Korean J Phys Educ. 2023;62:529–41.

[47] Kwon JA Jang JJ. Factors influencing injury severity and frequency among Korean sports participants in their 20s and 30s. Healthcare (Basel). 2024;12:664. 38540628 10.3390/healthcare12060664 PMC10970244

[48] Jo KH Lee SM So WY Lee EJ. Mediating effect of sports safety awareness between sports activity habits and the intention to complete safety education among Korean adolescents. Healthcare. 2024;11:1891. 10.3390/healthcare11131891 PMC10341132 37444725

